# ZEB2 upregulation modulates the polarization of TAMs toward the immunosuppressive state in EGFR-TKI-resistant NSCLC

**DOI:** 10.20517/cdr.2024.206

**Published:** 2025-05-29

**Authors:** Yunhuan Liu, Yong Yu, Congli Hu, Minlin Jiang, Chao Zhao, Xuefei Li, Lei Cheng, Caicun Zhou

**Affiliations:** ^1^Department of oncology, Shanghai Pulmonary Hospital Affiliated to Tongji University, Shanghai 200433, China.; ^2^Department of oncology, Shanghai East Hospital Affiliated to Tongji University, No.1800 Yuntai Road, Pudong New District, Shanghai 200120, China.; ^3^Department of Radiology, Yancheng TCM Hospital Affiliated to Nanjing University of Chinese Medicine, Yancheng 224002, Jiangsu, China.; ^#^Authors contributed equally.

**Keywords:** EGFR-TKI resistance, tumor-associated macrophages (TAMs), ZEB2, tumor microenvironment (TME), immunotherapy resistance, PI3K-Akt pathway

## Abstract

**Aim:** Immune checkpoint inhibitors (ICIs) have revolutionized the treatment approach for NSCLC. However, the effectiveness of ICI therapy in patients with EGFR-driven NSCLC, particularly those resistant to EGFR-TKI, has been disappointing. The immunosuppressive tumor microenvironment (TME) following EGFR-TKI therapy has been proved to significantly affected the effectiveness of ICIs. Therefore, studying the mechanism behind the development of a suppressive TME and exploring potential interventions is crucial for research on EGFR-TKI-resistant NSCLC.

**Methods:** ZEB2 levels were quantified in human NSCLC cell lines and in tumor specimens from NSCLC patients by quantitative RT-PCR (qRT-PCR), WB, and immunohistochemical staining. To examine how ZEB2 affected macrophage polarization, M1/M2 marker profiles were measured with qRT-PCR and flow cytometry. Changes in cytokine production triggered by altered ZEB2 expression were determined with qRT-PCR, ELISA, and Meso Scale Discovery electrochemiluminescence assays. The direct binding of ZEB2 to cytokine-gene promoters was tested using a dual-luciferase reporter system. Upstream regulatory pathways were investigated by correlating LUAD transcriptomic data from TCGA with ZEB2 expression and validating key findings via western blotting. Finally, cell-derived xenograft (CDX) models were generated by subcutaneously implanting pre-treated PC9 or HCC827 cells into BALB/c nude mice to verify the impact of EGFR-TKI resistance and ZEB2 on tumor-associated macrophage (TAM) polarization *in vivo*.

**Results:** It was elucidated that EGFR-TKI resistance upregulated the M2 polarization biomarkers, Arg-1 (PC9-GR: *P* < 0.01; HCC827-GR: *P* < 0.05) and IL4 (PC9-GR: *P* < 0.01; HCC827-GR: *P* < 0.01), while downregulated the M1 polarization biomarkers, TNF-α (PC9-GR: *P* < 0.01; HCC827-GR: *P* < 0.01), IL1β (PC9-GR: *P* < 0.01; HCC827-GR: *P* < 0.01), and IL6(PC9-GR: *P* < 0.001; HCC827-GR: *P* < 0.001) in NSCLC cell lines. Meanwhile, CD206^+^ TAMs (PC9-GR: *P* < 0.05; HCC827-GR: *P* < 0.01) were increased and CD86^+^ TAMs (PC9-GR: *P* < 0.05; HCC827-GR: *P* < 0.05) were decreased in both EGFR-TKI-resistant mice models. Apart from the formation of suppressive TME, ZEB2 was found to be upregulated in PC9-GR (qRT-PCR: *P* < 0.0001; WB: *P* < 0.05) and HCC827-GR (qRT-PCR: *P* < 0.0001; WB: *P* < 0.05) cells. The same trend was also noticed in clinical samples, with ZEB2 upregulated after gefitinib resistance in NSCLC patients (*P* < 0.0001). Based on these findings, ZEB2 knockdown was proved to downregulate Arg-1 (PC9-GR: *P* < 0.01; HCC827-GR: *P* < 0.05) and IL4 (PC9-GR: *P* < 0.01; HCC827-GR: *P* < 0.001), while upregulate the TNF-α (PC9-GR: *P* < 0.0001; HCC827-GR: *P* < 0.0001), IL1β (HCC827-GR: *P* < 0.001), and IL6 (PC9-GR: *P* < 0.01; HCC827-GR: *P* < 0.001), indicating its role in M1/M2 polarization in both EGFR-TKI-resistant NSCLC cell lines. The downregulation of CD206^+^ TAMs (PC9-GR: *P* < 0.05; HCC827-GR: *P* < 0.01) and the upregulation of CD86^+^ TAMs (PC9-GR: *P* < 0.001; HCC827-GR: *P* < 0.05) also demonstrated the reversion of suppressive TME after ZEB2 knockout in EGFR-TKI-resistant mice models. Additionally, after the intervention of MK2206, which was an Akt inhibitor, ZEB2 expression was suppressed at both low (PC9-GR: *P* < 0.001; HCC827-GR: *P* < 0.001) and high concentrations (PC9-GR: *P* < 0.001; HCC827-GR: *P* < 0.0001). Finally, the mechanism underlying ZEB2’s regulation on TAM polarization was proved to be associated with cytokine secretion. According to the results of ELISA, apart from its inducement on TGF-β1 secretion (PC9-GR: *P* < 0.0001; HCC827-GR: *P* < 0.0001), ZEB2 could directly bind to the promoter region of CSF-1 to elevate its secretion (PC9-GR: *P* < 0.0001; HCC827-GR: *P* < 0.0001).

**Conclusion:** In EGFR-TKI-resistant NSCLC, activation of the PI3K-Akt cascade drove a marked rise in ZEB2 expression. The elevated ZEB2 increased CSF-1 and TGF-β1 release, steering macrophages toward an M2 phenotype while impeding M1 polarization. Accordingly, suppressing ZEB2 had the potential to reshape the TME and enhance the effectiveness of ICIs once EGFR-TKI resistance had emerged.

## INTRODUCTION

Lung cancer remains the leading contributor to cancer-related mortality worldwide, with approximately 2.5 million cases reported in the latest Cancer Statistics from the American Cancer Society (ACS)^[1]^. Non-small cell lung cancer (NSCLC) accounts for nearly 80% of all newly diagnosed lung tumors and is notoriously difficult to identify at an early stage. As a result, most NSCLC cases are detected only after the disease has progressed, yielding a persistently low five-year survival rate^[2]^.

In recent years, targeted therapy has significantly improved the treatment and prognosis of advanced non-small cell lung cancer (NSCLC). Among these therapies, tyrosine kinase inhibitors of epidermal growth factor receptors (EGFR-TKIs), effective in 40%-60% of Asian patients with driver gene mutations, have revolutionized treatment outcomes^[3]^. However, resistance to EGFR-TKIs typically develops within 9 to 15 months of progression-free survival (PFS), leaving limited therapeutic options for patients with resistance^[4]^. Immune checkpoint inhibitors (ICIs), particularly those targeting PD-1/PD-L1, have gained prominence in cancer therapy, demonstrating efficacy in various solid tumors. In advanced NSCLC, ICIs have been integrated into first-line treatment regimens^[5]^. Despite their success in other cancers^[6,7]^, the efficacy of ICIs in EGFR-TKI-resistant NSCLC remained limited. Large-scale phase III trials, such as CheckMate 722 and Keynote 789, showed no improvement in PFS or overall survival (OS) with the combination of immunotherapy and chemotherapy compared to platinum-based chemotherapy in EGFR-TKI-resistant patients^[8,9]^. Furthermore, a 2020 WCLC clinical trial reported that only 1 out of 31 EGFR-TKI-resistant patients achieved a partial response with nivolumab and ipilimumab combination therapy, suggesting dual immunotherapy may not be optimal for these patients^[10]^. Therefore, understanding the mechanisms behind ICIs’ limited efficacy in EGFR-TKI-resistant NSCLC and exploring strategies to enhance their effectiveness remained key research priorities.

Tumor microenvironment (TME)^[11-16]^, somatic mutations in tumors, copy number variants^[17,18]^, and tumor immunogenicity^[19-21]^ were all proved in previous studies to be associated with the therapeutic effect of ICIs. Immunosuppressive TME, symbolizing by impeding T cell infiltration and incomplete cytotoxicity, suppressive tumor-associated macrophage (TAM) and regulatory T cell (Treg) recruitment, as well as inhibitory cytokines production, was related to EGFR-TKI resistance in multiple studies^[22,23]^. An earlier work of our team using a murine model attempted to elucidate the changes of TME during the course of EGFR-TKIs treatment. At the early stage of EGFR-TKI usage, we observed a short-term inhibition of tumor cell growth, an increased presence of CD8+ T cells, DCs and M1-like TAMs, along with impeding Treg infiltration and M2 polarization. However, the depletion of antitumor effector cells and upregulation of IL10 and CCL2 in serum, which could induce a suppressive immune microenvironment, occurred at a later stage of treatment^[24]^. The evidence above suggested that EGFR-TKI resistance could influence the efficacy of ICI therapy by modulating the TME.

Macrophage, one of the most abundant immune cells in the TME, was elucidated to promote tumor progression and affect the therapeutic efficacy of ICIs via several mechanisms, such as cytokine/chemokine production, the induction of chronic inflammation, angiogenesis, and immunosuppression^[25]^. Macrophages vary in a spectrum between “M1 phenotype”, which is pro-inflammatory, and “M2 phenotype”, which is anti-inflammatory. Previous studies indicated that oncogenic driver mutations in tumor cells led to M2 polarization of TAMs and then created an immunosuppressive microenvironment^[26,27]^. The pro-inflammatory M1-TAMs were increased during the early stage of TKI usage, along with elevated antigen-presenting capacity in EGFR^L858R^-mutant tumors^[28]^. Meanwhile, it was proved that TKI treatment reduced the recruitment of M2 TAMs in multiple mouse models. There were several explanations for this phenomenon, such as decreasing proliferation of M2 TAMs, inhibition of M2 polarization of TAMs, and switching from M2 to M1 TAMs^[24,29,30]^. After TKI resistance happened, more TAMs infiltrating the lesions were observed in biopsies by single-cell RNA sequencing analysis^[31]^, while the changes in the subtypes of TAMs had not been fully clarified.

ZEB2, a 2-handed zinc finger/homeodomain protein, has indispensable functions during early fetal development and cancer progression. Owing to its high homology with ZEB1, it engaged paired E-box elements through zinc-finger domains at both termini^[32]^. Human Protein Atlas data showed strong ZEB2 expression in neural and lymphoid tissues; heterozygous loss-of-function variants caused Mowat-Wilson syndrome, manifested by intellectual disability, characteristic facies, epilepsy, and a predisposition to Hirschsprung disease^[33,34]^. Extensive work has since linked ZEB2 to lymphocyte maturation, including its regulation of age-associated B cells^[35]^, age-associated T cells^[36]^, and CD11c^+^ B cell subsets^[37]^. As a canonical EMT driver, ZEB2 also promoted tumor progression and dissemination: its levels tracked with TNM stage in colorectal carcinoma^[38]^ and portended poor prognosis in urogenital cancers^[39]^. Yet the detailed mechanisms connecting ZEB2 to tumor evolution and therapy resistance, particularly its influence on the tumor microenvironment (TME), remained unresolved in NSCLC.

Here, we concentrated on EGFR-TKI-resistant NSCLC to delineate how ZEB2 remodeled the TME, with emphasis on tumor-associated macrophage (TAM) dynamics. By mapping the signaling pathways that couple drug resistance to immune suppression, we aimed to identify ZEB2-directed interventions capable of amplifying immunotherapy efficacy in these patients.

## METHODS

### Patients’ tumor samples

Ten NSCLC patients who underwent tumor biopsies at Shanghai Pulmonary Hospital, Tongji University, between December 2020 and October 2023, were prospectively enrolled. Written informed consent was obtained from each participant. Matched biopsies taken before and after the development of EGFR-TKI resistance were collected. Resistance to the first-generation EGFR-TKI gefitinib was verified through mutation testing and subsequent clinical follow-up. The protocol (approval No. K21-313Z) was cleared by the Shanghai Pulmonary Hospital Ethics Committee. Baseline demographic and clinical data were listed in Supplementary Table 1. Tumor specimens were formalin-fixed, paraffin-embedded, and sliced into 4 µm sections for downstream analyses.

### Cell lines

The NSCLC lines PC9 and HCC827, together with the human monocytic line THP-1, were obtained from the Cell Resource Center, Chinese Academy of Sciences (Shanghai, China). Both PC9 and HCC827 harbor EGFR exon-19 deletions. PC9 and HCC827 cells were maintained in high-glucose DMEM containing 10% fetal bovine serum (FBS), 1% penicillin-streptomycin, and 0.5 µg/mL mycoplasma removal agent (Beyotime, C0288M). THP-1 cells were propagated in RPMI-1640 supplemented with the same concentrations of FBS, antibiotics, and mycoplasma reagent. For macrophage differentiation, THP-1 cultures were exposed to 200 ng/mL phorbol 12-myristate 13-acetate (PMA; MedChemExpress, HY-18739) for 48 h to yield M0 macrophages, which were subsequently used in all downstream assays.

### Generation of gefitinib-resistant PC9 (PC9-GR) and HCC827 (HCC827-GR) cells

Gefitinib-resistant PC9 (PC9-GR) and HCC827 (HCC827-GR) cells were established through long-term exposure to increasing concentrations of gefitinib. Initially, both PC9 and HCC827 cells were cultured in high-glucose DMEM supplemented with 10% fetal bovine serum (FBS), 1% penicillin-streptomycin antibiotic solution, and 0.5 μg/mL mycoplasma removal agent. The cells were exposed to gefitinib starting at a low concentration, and the concentration was gradually increased in a stepwise fashion every 1-2 weeks. The gefitinib concentration was progressively escalated until it reached 2 μM, at which point the cells were continuously cultured in this concentration. During this period, cell proliferation rates and morphology were carefully monitored under a light microscope. Once the cells exhibited growth rates and morphology similar to those of the untreated parental cells, they were considered resistant. This process of resistance development took more than three months, during which the cells adapted to the selective pressure of gefitinib. To maintain the resistance phenotype, both PC9-GR and HCC827-GR cells were routinely cultured in high-glucose DMEM supplemented with 10% FBS, 1% penicillin-streptomycin, and 0.5 μg/mL mycoplasma removal agent, with the maintained gefitinib concentration of 2 μM. To confirm the establishment of resistance, cell viability was assessed using a cell counting kit-8 (CCK-8, Beyotime, C0038) according to the manufacturer’s instructions. A significant reduction in viability was observed in untreated parental cells when exposed to gefitinib, whereas gefitinib-resistant cells (PC9-GR and HCC827-GR) demonstrated significantly higher resistance to gefitinib-induced growth inhibition, confirming the successful establishment of resistance. This method of generating gefitinib-resistant cell lines was a robust model for studying EGFR-TKI resistance mechanisms in NSCLC.

### Generation of human TAMs

Peripheral blood donated by healthy volunteers was processed with Ficoll-Paque density gradients to isolate peripheral blood mononuclear cells (PBMCs). CD14^+^ monocytes were positively selected from the PBMC fraction using the EasySep Human CD14 kit (StemCell Technologies, 17858) and shown by flow cytometry to exceed 97% purity. Monocytes were differentiated for seven days in RPMI-1640 supplemented with 10% FBS, 1% penicillin-streptomycin, 0.5 µg/mL mycoplasma removal agent, and 100 ng/mL recombinant human M-CSF (BioLegend, 574806). Tumor-associated macrophages (TAMs) were generated by incubating the resulting macrophages for 24 h in a 1:1 mixture of conditioned medium from EGFR-TKI-sensitive or -resistant NSCLC cells and fresh RPMI-1640 containing 10% FBS.

### Preparation of conditional medium (CM)

PC9-GR and HCC827-GR cells were transfected with ZEB2-targeting siRNAs (RiboBio, siBDM2500) and incubated for 72 h. The cultures were then switched to serum-free high-glucose DMEM supplemented with 1% penicillin-streptomycin and 0.5 μg/mL mycoplasma inhibitor for a further 24 h. The conditioned medium was collected and clarified by centrifugation at 2,500 rpm for 20 min, and the supernatant was applied to M0 THP-1 macrophages for 24 h to induce polarization.

### IHC staining and quantification

Formalin-fixed lung-tumor specimens were paraffin-embedded and sectioned at 4 µm. Slides were dewaxed in xylene, re-hydrated through graded ethanols, and subjected to heat-mediated antigen retrieval in pH 6.0 citrate buffer at 100 °C. After cooling to room temperature, sections were rinsed three times in PBS (5 min each) and incubated in 3% H_2_O_2_ for 25 min in the dark to quench endogenous peroxidase. Sample areas were outlined, and nonspecific binding was blocked with 3% BSA for 30 min at ambient temperature. Primary antibodies, anti-ZEB2 (1:100, Cusabio, CSB-PA026425DSR2HU) and anti-CD206 (1:300, Servicebio, GB115273-100), were applied overnight at 4 °C in a humid chamber. Following three PBS washes, sections were exposed to HRP-labeled goat anti-rabbit IgG (1:500, Servicebio, GB23303) for 50 min at room temperature. The signal was developed with 3,3’-diaminobenzidine (DAB; Beyotime, P0202) according to the manufacturer’s instructions.

More than 5 fields for each slide were captured at × 400 magnification and the analysis was conducted by two independent, professional investigators. The immunoreactivity of each biomarker was assessed via a semi-quantitative scoring system: 0 for < 5% positive cells, 1 for 5%-25% positive cells, 2 for 25%-50% positive cells, 3 for 50%-75% positive cells, and 4 for > 75% positive cells. The level of staining intensity was also evaluated through the semi-quantitative scoring system presented in the following: 0 for negative, 1 for weak staining, 2 for moderate staining, and 3 for strong staining. The final score of each sample was noted as the product of the score of immunoreactivity and the score of staining intensity. The median value was used to assess the expression level of each biomarker in each sample. The number of positive cells in each field was also counted at × 400 magnification.

### ZEB2 knockout and knockdown

Lentiviral vectors for ZEB2 gene disruption were obtained from QEgene Inc. NSCLC cells were seeded in 6-well plates and allowed to adhere for 24 h until 60% confluence. Infection was carried out by replacing the culture medium with 1 mL fresh medium containing 5 µg/mL polybrene and the lentivirus at the manufacturer-recommended multiplicity of infection (MOI). After 48 h, transduced populations were enriched with 3 µg/mL puromycin; single-clone selection was maintained for approximately two months. Knockout efficiency was verified by qRT-PCR and immunoblot analysis.

For transient silencing experiments, ZEB2 expression in PC9-GR and HCC827-GR cells was reduced using a RiboBio siRNA transfection kit (RiboBio, Guangzhou, China) according to the supplier’s protocol. Three target sequences were utilized:

si-h-ZEB2_001: GGAGTTACTTCTCCTAATA

si-h-ZEB2_002: GAAGCTACGTACTTTAATA

si-h-ZEB2_003: GCACTAGTCCCTTTATGAA

A non-targeting control siRNA supplied in the kit served as the negative control. Silencing efficiency was confirmed by qRT-PCR and western blotting.

### Reagents used in experiments

To assess the activation of the PI3K-Akt, MAPK, and NF-κB signaling pathways, AKT1/2/3 inhibitor MK2206 (MedChemExpress, HY10358), ERK 1/2 inhibitor U0126 (MedChemExpress, HY12031A), and NF-κB inhibitor PDTC (MedChemExpress, HY18738) were used respectively on resistant NSCLC cells for 72 h. Then, the mRNA and protein of these cells were harvested for the following experiments.

### RNA extraction and quantitative real-time PCR (qRT-PCR)

Total RNA was extracted from treated NSCLC cells and TAMs with the RNAfast2000 kit (Fastagen, 220011) in accordance with the manufacturer’s protocol. After quantification, 500 ng of RNA was reverse-transcribed using Evo M-MLV RT Premix for qPCR (Accurate Biotechnology, AG11796). Transcript levels were determined by SYBR Green-based real-time PCR with Premix Pro Taq HS (Accurate Biotechnology, AG11718). Relative expression was calculated by the ΔCT approach and normalized to GAPDH. All assays were conducted in triplicate. Primer sequences were provided in Supplementary Table 2.

### Western blotting

The sensitive and resistant NSCLC cells after treatment were harvested. The lysis solution containing RIPA lysis buffer (Epizyme, PC101), protease inhibitor (Epizyme, GRF101), and phosphatase inhibitor (Epizyme, GRF102) was added according to the amount of cells. BCA Protein Assay Kit (Epizyme, ZJ102) was used to determine the concentration of protein in each sample. After the protein concentration was adjusted to the same, 15 μL protein samples were added on 7.5% PAGE Gels (Epizyme, PG211) and transferred to PVDF membranes. The PVDF membranes were blocked by rapid blocking buffer (Epizyme, PS108P) for 1 h according to the manufacturer’s instructions and then incubated with specific primary antibodies at 4 °C overnight. The following primary antibodies were used in this research: anti-ZEB2 (1:100, ABclonal,A5705), anti-Akt1/2/3 (1:1,000, ABclonal, 18675), anti-Phospho-Akt-S473 (1:500, ABclonal, AP1208), anti-ERK1/2 (1:500, ABclonal, A4782), anti-Phospho-ERK1-T202+ERK2-T185 (1:500, ABclonal, AP0485), anti-p65/RelA (1:500, ABclonal, A19653), and anti-Phospho-p65/RelA-S536 (1:1,000, ABclonal, AP1294). After this process, the membranes were rinsed in 1 × TBST (Epizyme, PS103S) 3 times and incubated with HRP-conjugated goat anti-rabbit secondary antibody (1:2,000, ABclonal, AS014). Then, the protein bands were visualized using the ECL chemiluminescent detection kit (Beyotime, P00018AS) on the Amersham Imager 600 machine.

### Enzyme-linked immunosorbent assay (ELISA)

Culture supernatants from treated NSCLC cells were cleared by centrifugation (2,500 rpm, 20 min) and analyzed for M-CSF and TGF-β1 using ABclonal ELISA kits RK00044 and RK00055, respectively, in accordance with the manufacturers’ protocols. Briefly, 100 µL of standards or suitably diluted samples were loaded onto pre-coated plates and incubated for 2 h at room temperature. After washing, the biotin-labeled detection antibody was applied for 1 h, followed by a 30 min streptavidin-HRP step. Color was developed with TMB substrate in the dark for 20 min, and absorbance at 450 nm was measured on a SpectraMax ABS Plus microplate reader.

### Meso scale discovery (MSD) electrochemiluminescence

Concentrations of human IL4, CXCL9, and CCL8 in the supernatant of NSCLC tumor cells were detected using the MSD U-plex flexible personalized multiplexing kit. 25 μL/well standards or samples were loaded into the pre-coated U-plex plates. After incubating for 1 h, the plates were rinsed 3 times. 50 μL detection antibody was added into each well and incubated for another 1 h. Finally, 150 μL 2 × read buffer T was added for detection on the MSD machine (SQ120MM).

### Flow cytometry analysis of the percentages of TAMs and M1/M2 TAMs

Tumor tissues harvested from mouse models were dissociated and incubated in the enzymatic cocktail containing 2.2 mL DMEM, 100 uL Enzyme H, 10 uL Enzyme R, and 12.5 uL Enzyme A using the gentleMACS^TM^ Octo Dissociator with heaters according to manufacturer’s guidance (Miltenyi Biotec, 130-095-929). The dissociated tissues were filtered through the 70 μm strainers (Miltenyi Biotec, 130098462) to remove the large debris. The THP-1 cells, after the intervention, were suspended in PBS for incubation. The cell suspensions were diluted to 1 × 10^6^/100 μL and incubated with antibodies. The antibodies used in this experiment were presented as the following: APC-Cy7 Rat Anti-Mouse CD45(30-F11) (BD Pharmingen, 557659), FITC Rat Anti-CD11b(M1/70) (BD Pharmingen, 557396), BV650 Rat Anti-Mouse F4/80(T45-2342) (BD Pharmingen,743282), BV605 Rat Anti-Mouse CD86(GL1) (BD Pharmingen, 563055), PE Rat Anti-Mouse CD206(Y17-505) (BD Pharmingen, 568273), APC Mouse Anti-Human CD86(2331 (FUN-1)) (BD Pharmingen,555660), PE Mouse Anti-Human CD206(19.2) (BD Pharmingen, 555954), and Fixable Viability Stain 510 (BD Pharmingen, 564406). The percentages of TAMs and M1/M2 TAMs were assessed with a FACS (Beckman Coulter, California, the United States of America).

### Dual-luciferase reporter assay

To examine whether ZEB2 directly regulated cytokine promoters, wild-type promoter segments from CSF-1, CCL8, and IL-4, all known to drive TAM recruitment and polarization, were inserted into the dual-luciferase reporter vector GV534 (GeneChem, Shanghai). A ZEB2 expression construct was generated in GV712 (GeneChem). HEK-293T cells were seeded in 24-well plates at 1 × 10^5^ cells well^-1^ and cultured at 37 °C until 60% confluence. Five experimental conditions were established, combining GV534-Cytokine-WT, GV712-ZEB2, and their respective empty vectors; CV045 (GeneChem) carrying Renilla reniformis luciferase served as the internal control. Transfections were performed with Lipofectamine 2000 (Invitrogen, 11668019) following the vendor’s protocol. After 48 h, firefly and Renilla activities were quantified with the Dual-Luciferase Reporter Assay System (Promega, E1910) on an Infinite 200 PRO reader (Tecan). Each experiment was independently repeated three times.

### Bioinformatic analysis

The RNA-seq dataset GSE121634 (Illumina HiSeq 4000, platform GPL20301) was downloaded from GEO and used to compare transcriptomic profiles of NSCLC cell lines before and after the acquisition of erlotinib resistance^[40]^. Differential expression between drug-sensitive and -resistant variants of HCC827 and HCC4006 was determined with the DESeq2 package. Associations between ZEB2 and key signaling pathway markers or cytokines were evaluated in the TCGA lung adenocarcinoma (LUAD) cohort by R-based analysis and visualized with Chiplot^[41]^. ZEB2 expression was then correlated with the abundance of immune-cell subsets in LUAD using TIMER 2.0 and the CIBERSORT-ABS deconvolution method^[42]^. Finally, ZEB2-related gene set enrichment was examined through the GSEA module of the LinkedOmics web platform.

### *In vivo* studies

Female BALB-c nude mice aged 6-8 weeks were purchased from SLAC Animal (Shanghai, China) and housed in the SPF-level animal center. All procedures on animals were approved by the Institutional Committee for Animal Care and Use, Shanghai Pulmonary Hospital, and followed current standards of animal use in China. PC9 and PC9GR with or without ZEB2 knockout, HCC827 and HCC827-GR with or without ZEB2 knockout were inoculated into the right flanks of mice subcutaneously at a concentration of 1 × 10^6^ cells/100 μL. The weights of mice and the sizes of tumors were closely monitored and recorded during the experiment. The tumor size was calculated by the following formula: volume (mm^3^) = (length × width^2^) × 0.5.

### Statistical analysis

All quantitative results are presented as mean ± standard deviation (SD) or median with inter-quartile range (25th-75th percentile). Paired Student’s *t*‐tests were applied to matched clinical specimens. Unpaired Student’s *t*‐tests were used for two independent groups, whereas analyses involving three or more groups employed one-way ANOVA. Spearman rank coefficients quantified correlations between biomarker levels. Each experiment was performed in triplicate or more, and significance was set at *P* < 0.05 (^*^P < 0.05, ^**^P < 0.005, ^***^P < 0.0005, ^****^P < 0.0001). All statistical procedures were executed with GraphPad Prism 8.0.

## RESULTS

### M2 polarization of TAMs in NSCLC acquiring resistance to EGFR-TKI

**Figure 1:**
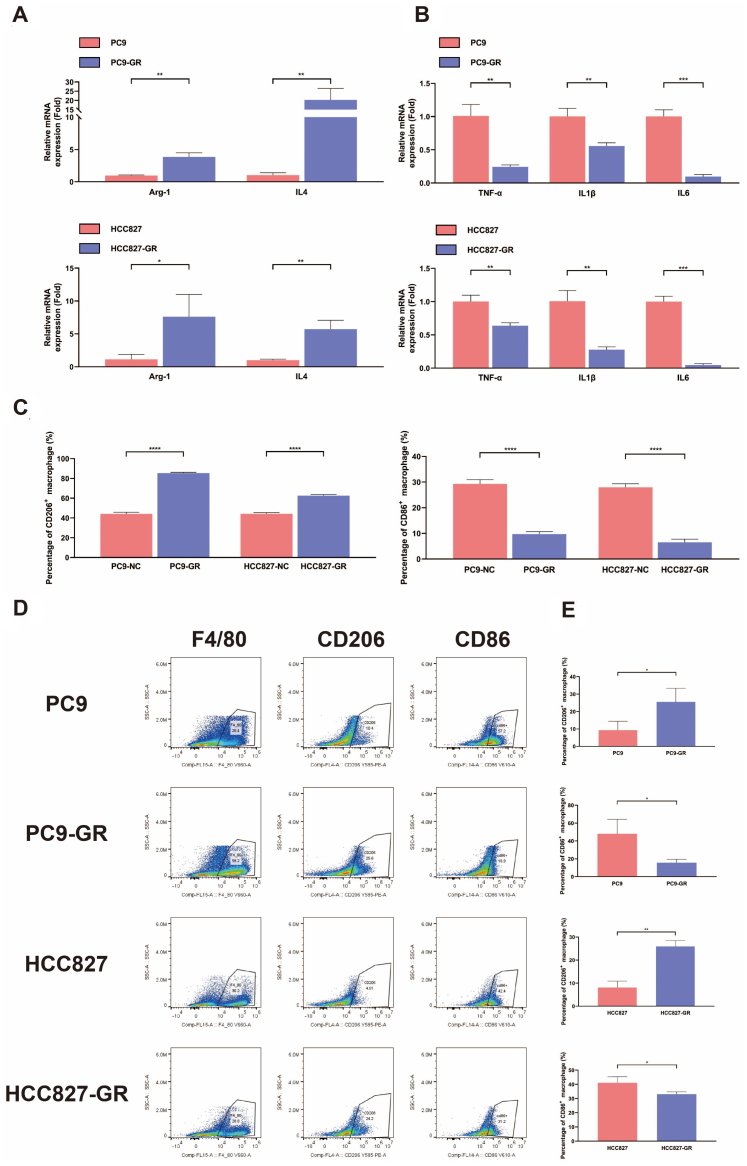
EGFR-TKI resistance induces M2 polarization and inhibits M1 polarization of TAMs in NSCLC. (A and B) qRT-PCR analysis of the expression M1/M2-like phenotypic biomarkers of THP-1 cells cultured with CM from EGFR-TKI-sensitive or -resistant PC9 and HCC827 cells. Data represent results from three independent experiments; (C) Flow cytometry analysis of the percentages of M1/M2-like phenotype macrophages in THP-1 cells intervened by CM from EGFR-TKI-sensitive or -resistant PC9 and HCC827 cells. Bar graphs show average values from three independent experiments; (D and E) The percentages of CD86^+^ and CD206^+^ TAMs in F4/80^+^ TAMs in tumor tissues from EGFR-TKI-sensitive or -resistant PC9 and HCC827 CDX mouse models were analyzed by flow cytometry; (D) Representative pseudo-color plots presenting the process of gating; (E) Average results from three independent experiments. ^*^P < 0.05, ^**^P < 0.005, ^***^P < 0.0005, ^****^P < 0.0001.

The polarization of TAMs in TME was determined both in vitro and *in vivo*. Using EGFR-TKI-sensitive and EGFR-TKI-resistant PC9 and HCC827 cells, CM was produced to induce M0 THP-1 cells according to the method described above. After 24 h of intervention, polarization of TAMs was detected via qRT-PCR and flow cytometry. The expression of TNF-α, IL1β, and IL6, which were usually used to detect the M1-like phenotype of TAMs, and the expression of Arg-1 and IL4, which were common biomarkers for the M2-like phenotype of TAMs, were determined by qRT-PCR. The results of this experiment showed that the expression of TNF-α, IL1β, and IL6 was suppressed in THP-1 cells after being stimulated by CM from PC9-GR and HCC827-GR, while the expression of Arg-1 and IL4 was elevated in the same groups [Figure 1A and B]. Compared to THP-1 cells stimulated with CM derived from EGFR-TKI-sensitive NSCLC cells, these changes indicated that M0 THP-1 cells exposed to CM from EGFR-TKI-resistant NSCLC cells were undergoing increased M2 polarization and suppressed M1 polarizations. The THP-1 cells were collected for flow cytometry after CM intervention to assess the percentages of M1 and M2 macrophages. The biomarker of M1 macrophage CD86 and the biomarker of M2 macrophage CD206 were used to assess M1 or M2 polarization. Consistent with the results of qRT-PCR, Figure 1C exhibits that CM from PC9-GR and HCC827-GR significantly increased the percentages of CD206^+^ macrophages in F4/80^+^ macrophages, while the percentages of CD86^+^ macrophages in F4/80^+^ macrophages were decreased by the same intervention. In addition, the influence of EGFR-TKI resistance on M1/M2 polarization was verified in mouse models. EGFR-TKI-sensitive and EGFR-TKI-resistant PC9 and HCC827 cells were subcutaneously inoculated into the right flanks of BALB/c nude mice. The tumors were harvested and the percentages of M1 and M2 TAMs were determined via flow cytometry. In both the tumors formed by PC9-GR and HCC827-GR inoculation, the percentages of CD206^+^ TAMs in F4/80^+^ TAMs were highly upregulated, while the percentages of CD86^+^ TAMs in F4/80^+^ TAMs were downregulated, suggesting that EGFR-TKI-resistant NSCLC cells could induce M2 polarization and inhibit M1 polarization in CDX mouse models [Figure 1D and E]. This trend was also reflected in the M1/M2 polarization of human TAMs. As shown in Supplementary Figure 1, conditional medium (CM) from PC9-GR and HCC827-GR cells could induce the upregulation of M2 phenotypic biomarkers, such as Arg-1 and IL4, while inhibiting the expression of M1 phenotypic biomarkers, including TNF-α, IL1β, and IL6. This trend was consistent with the status observed in the THP-1 cell line. Overall, these results elucidated that EGFR-TKI resistance could induce M2 polarization and inhibit M1 polarization of TAMs, which would contribute to the formation of a suppressive immune microenvironment in NSCLC.

### ZEB2 overexpression was observed in EGFR-TKI-resistant NSCLC

**Figure 2:**
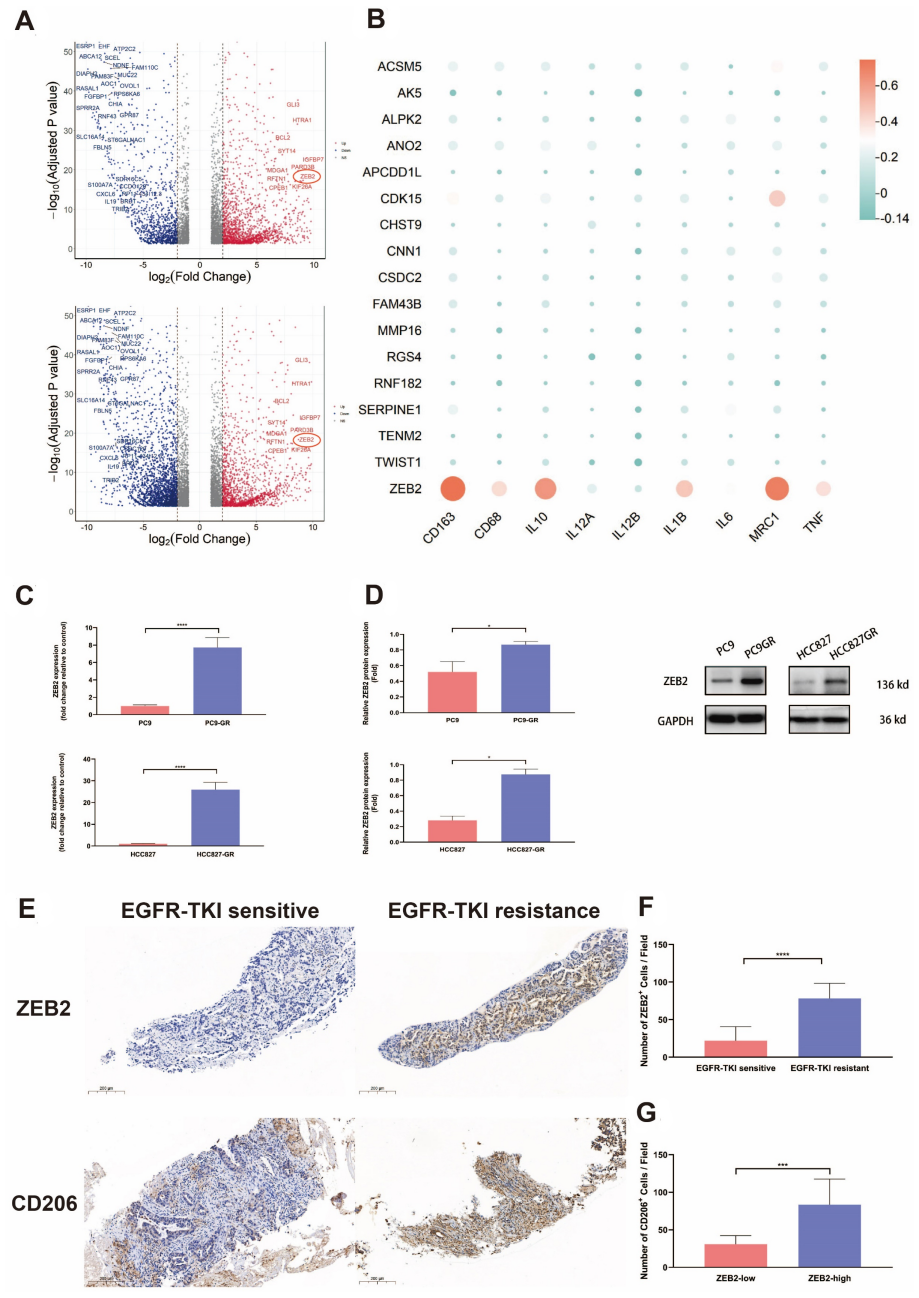
ZEB2 overexpression was observed in EGFR-TKI-resistant NSCLC. (A) Volcano plots demonstrating the DEGs in HCC827 and HCC4006 cells before and after erlotinib resistance, respectively. ZEB2 is circled in the plots; (B) Heatmap demonstrating the relations between the 17 most highly upregulated DEGs after EGFR-TKI resistance in HCC827 and HCC4006 cells and markers expressed by M1 (IL12, IL1β, TNF, IL6) and M2 (CD163, IL10, MRC1) macrophages; (C) qRT-PCR analysis to determine the mRNA expression of ZEB2 in EGFR-TKI-sensitive or -resistant NSCLC tumor cells. Data represent results from three independent experiments; (D) Western blotting analysis to determine the protein expression of ZEB2 in EGFR-TKI-sensitive or -resistant NSCLC tumor cells. Representative protein bands and the average results from 3 independent experiments are shown; (E) Representative IHC images of ZEB2 expression and CD206^+^ M2-like phenotype TAM infiltration. Brown granules denote positive staining. Scale bar: 200 μm; (F) Average counting numbers of ZEB2-positive tumor cells in each field (400 ×) of paired EGFR-TKI-sensitive and -resistant NSCLC samples; (G) Average counting numbers of CD206^+^ M2-like phenotype TAMs per field (400 ×) in NSCLC samples with high versus low ZEB2 expression. ^*^P < 0.05, ^***^P < 0.0005, ^****^P < 0.0001.

According to the transcriptomic analysis of EGFR-mutant NSCLC tumor cells HCC827 and HCC4006 before and after the development of EGFR-TKI resistance, 380 common upregulated DEGs (logFC > 2, adjusted *P* < 0.01) were selected [Figure 2A]. Among them, 17 most highly upregulated DEGs were included in the following assessment [Supplementary Tables 3 and 4]. ZEB2, among the 17 most highly upregulated DEGs, which was circled in Figure 2A, exhibited significant correlations with markers of M2 macrophage, such as CD163, MRC1, and IL10 [Figure 2B]. qRT-PCR and western blotting were conducted to determine both the mRNA and protein expression of ZEB2 in EGFR-TKI-sensitive or -resistant PC9 and HCC827 cells. Figure 2C and D denoted that ZEB2 was significantly upregulated in PC9-GR and HCC827-GR compared to corresponding EGFR-TKI-sensitive control groups. To further elucidate the level of ZEB2 expression in EGFR-TKI-resistant NSCLC, 10 patients undergoing biopsies at Shanghai Pulmonary Hospital with paired samples before and after the development of resistance to EGFR-TKI were recruited. The IHC staining of these samples illustrated that ZEB2-positive cells were markedly increased in patients’ samples after they developed resistance to EGFR-TKI [Figure 2E and F]. Broadly speaking, in NSCLC, ZEB2 overexpression was observed to be associated with EGFR-TKI resistance *in vitro, in vivo,* and *in silico*.

### ZEB2 upregulation in EGFR-TKI-resistant NSCLC tumor cells promoted M2 polarization and impeded M1 polarization

**Figure 3:**
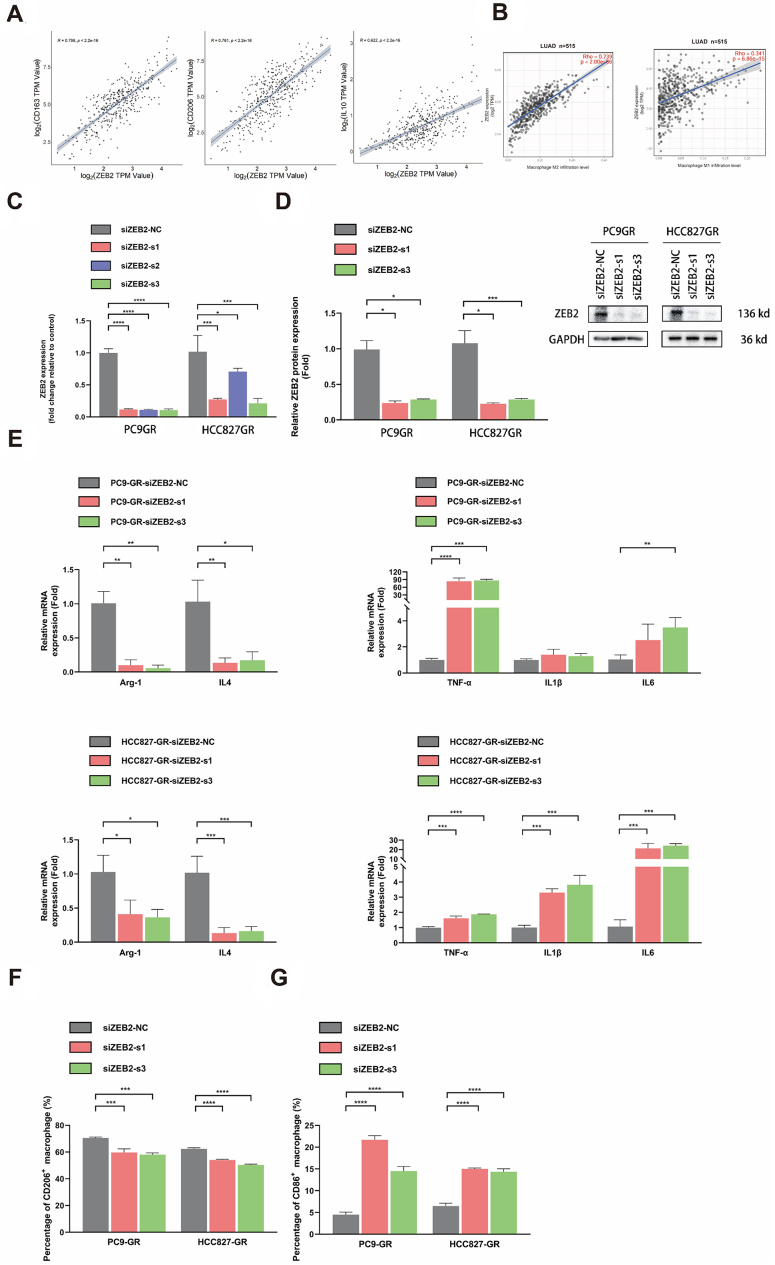
ZEB2 upregulation in EGFR-TKI-resistant NSCLC tumor cells promoted M2 polarization and impeded M1 polarization. (A) ZEB2 expression was positively correlated with the expression of CD163 (R = 0.798), CD206 (R = 0.761), and IL10 (R=0.622) in the LUAD cohort from the TCGA database; (B) Results of Timer 2.0 online analysis of the correlations between ZEB2 expression and M1 (R = 0.341) /M2 (R = 0.739) TAM infiltration in the LUAD cohort; (C and D) qRT-PCR and western blotting analysis to verify the efficiency of ZEB2 knockdown in PC9-GR and HCC827-GR cells. Data represent results from three independent experiments; (E) qRT-PCR analysis of the expression of M1/M2-like phenotypic biomarkers of THP-1 cultured with CM from EGFR-TKI-resistant PC9 and HCC827 cells with or without ZEB2 knockdown. Data represent results from three independent experiments; (F and G) Percentages of M1/M2-like phenotype macrophages in THP-1 cells after intervention by CM from PC9-GR and HCC827-GR cells with or without ZEB2 knockdown. Average results from three independent experiments are shown. ^*^P < 0.05, ^**^P < 0.005, ^***^P < 0.0005, ^****^P < 0.0001.

Based on the upregulation of ZEB2 after the formation of EGFR-TKI resistance and its correlations with M2 polarization demonstrated in Figure 2B, further research was conducted to reveal the role of ZEB2 in TAM polarization. First, a cohort of LUAD from the TCGA database was utilized to determine the relations between ZEB2 and characteristic markers of M2-like TAMs. ZEB2 expression had positive correlations with CD163 (R = 0.798), CD206 (R = 0.761), and IL10 (R = 0.622), with r values over 0.6 [Figure 3A]. It was also demonstrated in this cohort that ZEB2 expression had a strongly positive correlation with HAVCR2 (TIM-3) (R = 0.735), an immune checkpoint receptor that played a crucial role in regulating immune responses by promoting T cell exhaustion and limiting immune activation in NSCLC [Supplementary Figure 2]. Then, utilizing the website Timer 2.0, we explored the relationship between ZEB2 expression and M1/M2 TAM infiltration in the LUAD cohort with Cibersort-ABS for deconvolution. It was indicated in Figure 3B that ZEB2 was positively related to M2 TAM infiltration (R = 0.739) with a correlation coefficient r value over 0.6, while the correlation coefficient r value for correlation between ZEB2 and M1 TAM infiltration was 0.341.

**Figure 4:**
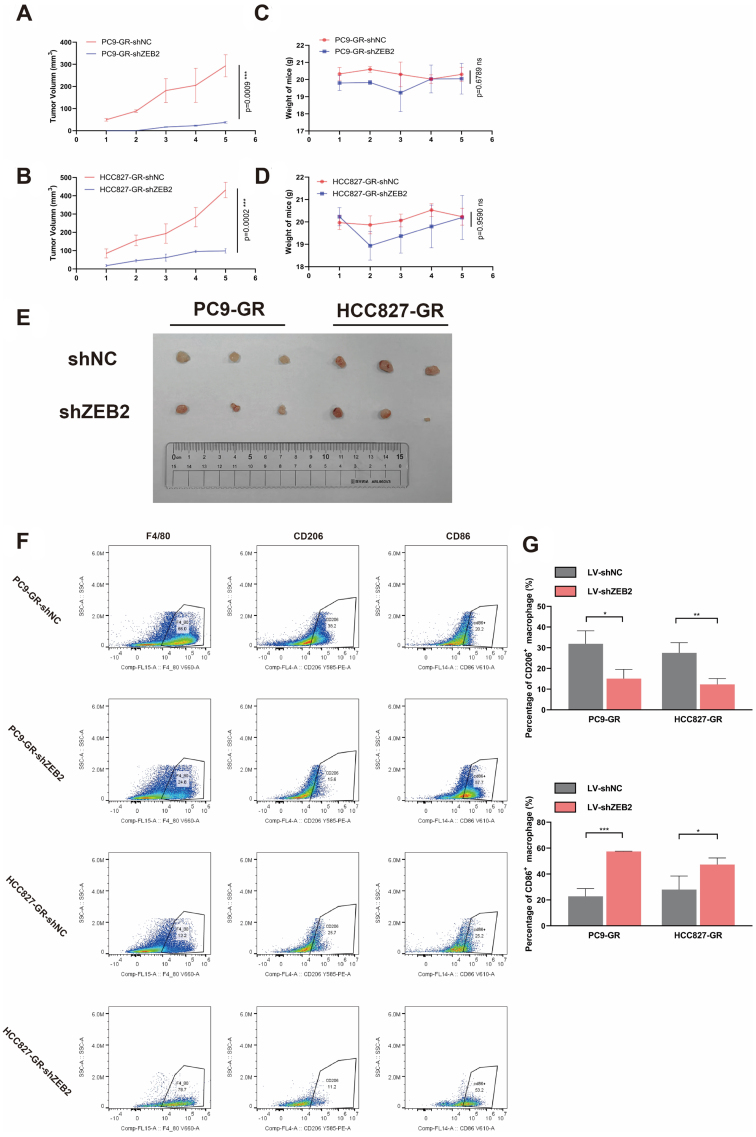
Flow cytometry results of THP-1 cells treated with CM from ZEB2 knockdown PC9-GR and HCC827-GR cells and tumor tissues from ZEB2 knockout PC9-GR or HCC827-GR CDX mouse models. (A and B) PC9-GR and HCC827-GR with or without ZEB2 knockout were inoculated into the right flanks of mice subcutaneously at a concentration of 1 × 10^6^ cells/100 μL. The sizes of tumors were measured every four days by caliper and five measurements during the experiment were presented as mean ± SD (*n* = 3 mice/group); (C and D) PC9-GR and HCC827-GR with or without ZEB2 knockout were inoculated into the right flanks of mice subcutaneously at a concentration of 1 × 10^6^ cells/100 μL. The weights of mice were recorded every four days and five results during the experiment were presented as mean ± SD (*n* = 3 mice/group); (E) Tumor images from each mouse are displayed; (F and G) The percentages of CD206^+^ and CD86^+^ TAMs in F4/80^+^ TAMs in tumor tissues from PC9-GR or HCC827-GR with or without ZEB2 knockout CDX mouse models were analyzed by flow cytometry; (F) Representative pseudo-color plots presenting the process of gating; (G) Average results from three independent experiments. ^*^P < 0.05, ^**^P < 0.005, ^***^P < 0.0005.

To further confirm the influence of ZEB2 on TAM polarization in EGFR-TKI-resistant NSCLC, ZEB2 in both PC9-GR and HCC827-GR cells was knocked down using siRNAs against ZEB2. The efficiency of knockdown was verified using qRT-PCR and western blotting. According to the results of these two experiments, siZEB2-s1 and siZEB2-s3 were selected for the following experiments due to their high efficiency of knockdown [Figure 3C and D]. The CM derived from PC9-GR and HCC827-GR, intervened by siZEB2, markedly downregulated the expression of M2-like phenotypic biomarkers Arg-1 and IL4. Meanwhile, it significantly elevated the expression of biomarkers characterizing M1 polarization, TNF-α, IL1β, and IL6 [Figure 3E]. Similarly, results from flow cytometry indicated that CM from PC9-GR and HCC827-GR with ZEB2 knockdown could significantly decrease the percentages of CD206^+^ macrophages and increase the percentages of CD86^+^ macrophages in F4/80^+^ macrophages [Figure 3F and G]. The impact of ZEB2 knockdown on M1/M2 polarization in EGFR-TKI-resistant NSCLC cell lines was also validated in human TAMs [Supplementary Figure 1]. In PC9-GR and HCC827-GR cells, ZEB2 knockdown reversed the EGFR-TKI-induced M2 polarization and suppression of M1 polarization in human TAMs. This led to the downregulation of M2 phenotypic biomarkers, such as Arg-1 and IL4, and the upregulation of M1 phenotypic biomarkers, including TNF-α, IL1β, and IL6. Both the outcomes of qRT-PCR and flow cytometry verified that ZEB2 knockdown could reverse the influence of EGFR-TKI resistance on TAM polarization, which meant that ZEB2 knockdown could reverse the inducement toward M2 polarization and inhibition of M1 polarization in TAMs caused by EGFR-TKI resistance. Additionally, we inoculated ZEB2 knockout PC9-GR and HCC827-GR, intervened by LV-shZEB2, into the BALB/c nude mice subcutaneously. To further evaluate whether ZEB2 knockout had any direct impact on the intrinsic properties of the tumor cells, such as cell viability, we performed CCK-8 assays on ZEB2 knockdown PC9-GR and HCC827-GR cells. As shown in Supplementary Figure 3, treatment with LV-shZEB2 did not result in a statistically significant decrease in the viability of either PC9-GR or HCC827-GR cells. These findings suggested that ZEB2 knockout did not substantially affect the cell viability of EGFR-TKI-resistant NSCLC cells. However, as displayed in Figure 4A and B, ZEB2 knockout could prominently reduce the growth rate of NSCLC tumors, while the weights of mice in PC9GR and HCC827GR groups with or without ZEB2 knockout showed no significant difference [Figure 4C and D]. Representative tumor images from each mouse are shown in Figure 4E. This observation indicated that the suppression observed in tumor growth after ZEB2 knockout was likely due to alterations in the immune microenvironment, rather than intrinsic changes in the tumor cells themselves. Consistent with this observation and the results of the in vitro study, ZEB2 knockout in TKI-resistant NSCLC tumor cells hindered the trend of M2 polarization in TAMs, demonstrated by lower percentages of CD206^+^ TAMs in F4/80^+^ TAMs, compared with TKI-resistant NSCLC cells treated with LV-shNC, while elevated the level of M1 polarization, evidenced by increasing percentages of CD86^+^ TAMs in F4/80^+^ TAMs [Figure 4F and G]. In addition, according to the results of IHC staining of patients’ samples in Figure 2E and G, patients with high ZEB2 expression in NSCLC tumor tissues exhibited remarkably increasing infiltration of CD206^+^ M2-like phenotype TAMs, which was a direct clinical evidence of the relationships between ZEB2 overexpression and M2 polarization of TAMs.

Overall, ZEB2 upregulation in EGFR-TKI-resistant NSCLC played a critical role in TAM polarization. Both in vitro and *in vivo* studies demonstrated that ZEB2 upregulation could promote M2 polarization and impede M1 polarization of TAMs, while ZEB2 knockout could reverse this effect on TAM polarization.

### ZEB2 expression was regulated via the PI3K-Akt pathway in EGFR-TKI-resistant NSCLC

**Figure 5:**
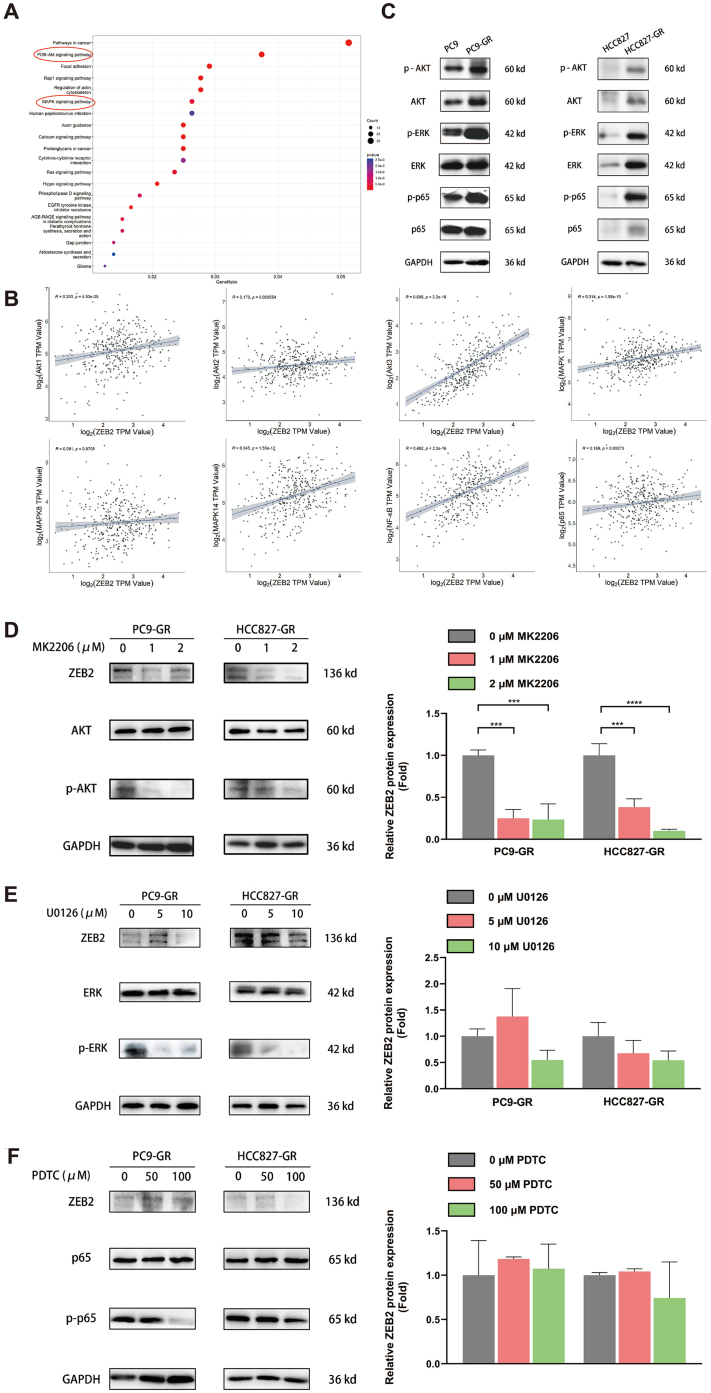
ZEB2 expression was regulated via the PI3K-Akt pathway in EGFR-TKI-resistant NSCLC. (A) The PI3K-Akt and MAPK signaling pathways were among the most significantly upregulated downstream pathways after EGFR-TKI resistance development in HCC827 and HCC4006 NSCLC cell lines. Common upregulated DEGs of these two cell lines before and after erlotinib resistance were included in this KEGG analysis; (B) The correlation between ZEB2 and key biomarkers of the PI3K-Akt [Akt1(R = 0.203), Akt2(R = 0.173), Akt3(R = 0.609)], MAPK [MAPK(R = 0.314), MAPK8(R = 0.091), MAPK14(R = 0.345)], and NF-κB signaling pathways [NF-κB(R = 0.482), p65(R = 0.169)] were assessed in the LUAD cohort from the TCGA database; (C) Western blotting was conducted to detect the phosphorylation level of key modulators of the PI3K-Akt, MAPK, and NF-κB signaling pathways before and after EGFR-TKI resistance in PC9 and HCC827 cells; (D-F) After 72 h of intervention with gradient concentrations of specific inhibitors of Akt(MK2206), ERK(U0126), and NF-κB(PDTC), western blotting was performed to determine the expression of ZEB2 in PC9-GR and HCC827-GR cells. Representative protein bands and the average results from 3 independent experiments are shown. ^***^P < 0.0005, ^****^P < 0.0001.

The upregulation of ZEB2 was proved to be associated with the development of EGFR-TKI resistance in NSCLC. Based on this result, the underlying mechanism of this relation needed to be explored. Thus, we analyzed the upregulated signaling pathway in HCC827 and HCC4006, which were EGFR-mutant NSCLC cell lines, before and after EGFR-TKI resistance development. The common upregulated DEGs of these two cell lines before and after erlotinib resistance were included in the Kyoto Encyclopedia of Genes and Genomes (KEGG) pathway enrichment analysis. The results are presented in Figure 5A. The PI3K-Akt and MAPK signaling pathways were among the most remarkably upregulated signaling pathways after the development of TKI resistance. According to previous research, NF-κB was also one of the classic altered signaling pathways after the development of TKI resistance in NSCLC^[43]^. The LUAD cohort from the TCGA database was assessed and the correlations between ZEB2 expression and the expression of key biomarkers of the PI3K-Akt, MAPK, and NF-κB signaling pathways, including Akt1/2/3, MAPK, JNK(MAPK8), p38(MAPK14), NF-κB, and p65, were determined. It was presented in Figure 5B that ZEB2 expression was positively significantly correlated with Akt3 expression, with the correlation coefficient r value over 0.6 (R = 0.609). Meanwhile, the r values of other correlations between ZEB2 and key modulators of signaling pathways were below 0.6. Built on the foundation of bioinformatic analysis, western blotting was used to confirm the influence of EGFR-TKI resistance on downstream signaling pathways, including the PI3K-Akt, MAPK, and NF-κB signaling pathways. Figure 5C shows that gefitinib resistance could increase the expression of p-Akt, p-ERK, and p-p65, which were phosphorylated classic biomarkers indicating the upregulation of these three signaling pathways, in both PC9-GR and HCC827-GR cells. To further confirm the effect of these downstream signaling pathways on ZEB2 expression, inhibitors of these pathways were used to treat TKI-resistant PC9 and HCC827 cells. MK2206, U0126, and PDTC were specific inhibitors of Akt, ERK, and NF-κB, respectively. We stimulated PC9-GR and HCC827-GR with gradient concentrations of these three inhibitors. The protein bands of western blotting and the semi-quantitative column plots are presented in Figure 5D-F. After MK2206 stimulation, the expression of ZEB2 was significantly decreased, denoting that Akt signaling pathway blockade could downregulate ZEB2 expression [Figure 5D]. However, the inhibition of ERK and NF-κB did not exhibit the same influence on ZEB2 expression [Figure 5E and F]. Figure 5E shows that although higher concentrations of U0126 (10 μM) induced a trend toward ZEB2 downregulation in both PC9-GR and HCC827-GR cell lines, this trend did not reach statistical significance. Moreover, at a lower concentration of U0126 (5 μM), a nonsignificant trend toward ZEB2 upregulation was noticed in the PC9-GR cell line. This suggested that the relationship between the MAPK signaling pathway and ZEB2 was likely not direct and could be influenced by multiple, complex factors, indicating that the MAPK signaling pathway may not be a primary regulator of ZEB2 expression in EGFR-TKI-resistant NSCLC cells. Furthermore, neither low (50 μM) nor high (100 μM) concentrations of PDTC, an NF-κB inhibitor, induced statistically significant changes in ZEB2 expression in both PC9-GR and HCC827-GR cells. This further suggested that the NF-κB pathway did not play a significant role in regulating ZEB2 expression in the context of EGFR-TKI resistance. Together, it was elucidated in this part that EGFR-TKI resistance could increase ZEB2 expression by upregulating the PI3K-Akt signaling pathway in NSCLC.

### ZEB2 promoted M2 polarization and impeded M1 polarization of TAMs via elevating the secretion of CSF-1 and TGF-β1

**Figure 6:**
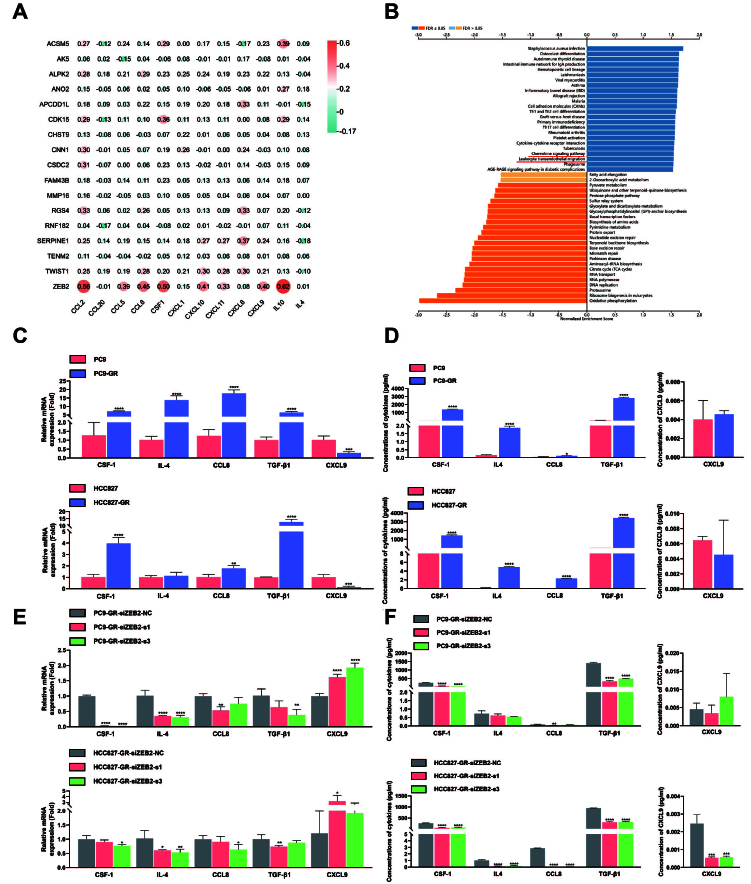
ZEB2 promotes M2 polarization and impedes M1 polarization of TAMs by elevating the secretion of CSF-1 and TGF-β1. (A) Heatmap demonstrating the relationships between 17 most highly upregulated DEGs after EGFR-TKI resistance in HCC827 and HCC4006 cells and cytokines that were proved to be associated with macrophage polarization; (B) A GSEA was conducted with the ZEB2-related genes in the TCGA LUAD dataset using the LinkedOmics online platform. The results of the KEGG pathway enrichment analysis showed that the chemokine signaling pathway and leukocyte transendothelial migration were among the upregulated pathways; (C) qRT-PCR was conducted to determine the alterations of cytokines associated with macrophage polarization after EGFR-TKI resistance in PC9 and HCC827 cells. Data represent results from three independent experiments; (D) Results of ELISA and MSD electrochemiluminescence of PC9 and HCC827 cells before and after EGFR-TKI resistance. The concentrations of TGF-β1 and CSF-1 were detected using ELISA, while the concentrations of CCL8, IL4, and CXCL9 were detected using MSD electrochemiluminescence. Data represent results from three independent experiments; (E) qRT-PCR analysis revealed that ZEB2 knockdown could reverse the alterations of CSF-1, TGF-β1, CCL8, IL4, and CXCL9 in PC9-GR and HCC827-GR cells. Data represent results from three independent experiments; (F) Results of ELISA and MSD electrochemiluminescence of PC9-GR and HCC827-GR cells with or without ZEB2 knockdown. The concentrations of TGF-β1 and CSF-1 were detected using ELISA, while the concentrations of CCL8, IL4, and CXCL9 were detected using MSD electrochemiluminescence. Data represent results from three independent experiments. ^*^P < 0.05, ^**^P < 0.005, ^***^P < 0.0005, ^****^P < 0.0001.

It was elucidated in several studies of TAM chemotaxis and polarization that various cytokines belonging to chemokine (C-C motif) ligands (CCLs), chemokine (C-X-C motif) ligands (CXCLs), and colony-stimulating factors (CSFs) could not only recruit TAMs but also induce M1/M2 polarization of TAMs^[44]^. In this research, we found that ZEB2 may be related to cytokine secretion through bioinformatic analysis. A total of 17 common, most highly upregulated DEGs in HCC827 and HCC4006 cell lines before and after erlotinib resistance were selected. The correlations between these 17 genes and cytokines related to TAM polarization were analyzed utilizing the LUAD cohort from the TCGA dataset. According to previous studies, 12 cytokines proved to be associated with macrophage polarization - CCL2^[45]^, CCL20^[46]^, CCL5^[47]^, CCL8^[48]^, CSF-1^[49]^, CXCL1^[50]^, CXCL10, CXCL11^[51]^, CXCL8^[52]^, CXCL9^[53]^, IL10, and IL4 - were included in this analysis. Additionally, TGF-β1, as a classic anti-inflammatory modulator, had been proved to be upregulated by ZEB2 in hepatocellular carcinoma according to previous research, so it was also included for further analysis^[54]^. Figure 6A suggests that ZEB2 was positively correlated with most of these cytokines among these 17 genes. In addition, the GSEA analysis demonstrated that the chemokine signaling pathway and leukocyte transendothelial migration were among the upregulated pathways based on the results of KEGG pathway enrichment [Figure 6B]. To explore the underlying mechanism of ZEB2 promoting M2 polarization and inhibiting M1 polarization in EGFR-TKI-resistant NSCLC, we hypothesized that ZEB2 could induce the polarization of TAMs by modulating the secretion of cytokines that were related to this process based on the results of bioinformatic analysis. The results of qRT-PCR showed that the expression of CSF-1, IL4, CCL8, TGF-β1, and CXCL9 were remarkably increased in PC9-GR and HCC827-GR compared with corresponding EGFR-TKI-sensitive control groups [Figure 6C]. Meanwhile, the secretion levels of these cytokines in the supernatant were assessed by ELISA or MSD electrochemiluminescence. Figure 6D shows that CXCL9 secretion was not significantly altered in two TKI-resistant NSCLC cell lines, while CSF-1, IL4, CCL8, and TGF-β1 secretions were markedly elevated in TKI-resistant PC9 and HCC827 cells. To consolidate the influence of ZEB2 on the secretion of CSF-1, IL4, CCL8, TGF-β, and CXCL9, we examined their expression and secretion in TKI-resistant NSCLC cells after ZEB2 knockdown. Figure 6E and F shows that ZEB2 knockdown significantly and consistently downregulated the expression and secretion of CSF-1, IL4, CCL8, and TGF-β1 in PC9-GR and HCC827-GR cells, while the alterations of the secretion of CXCL9 were not consistent with those of the mRNA expression of CXCL9 in the two cell lines.

**Figure 7:**
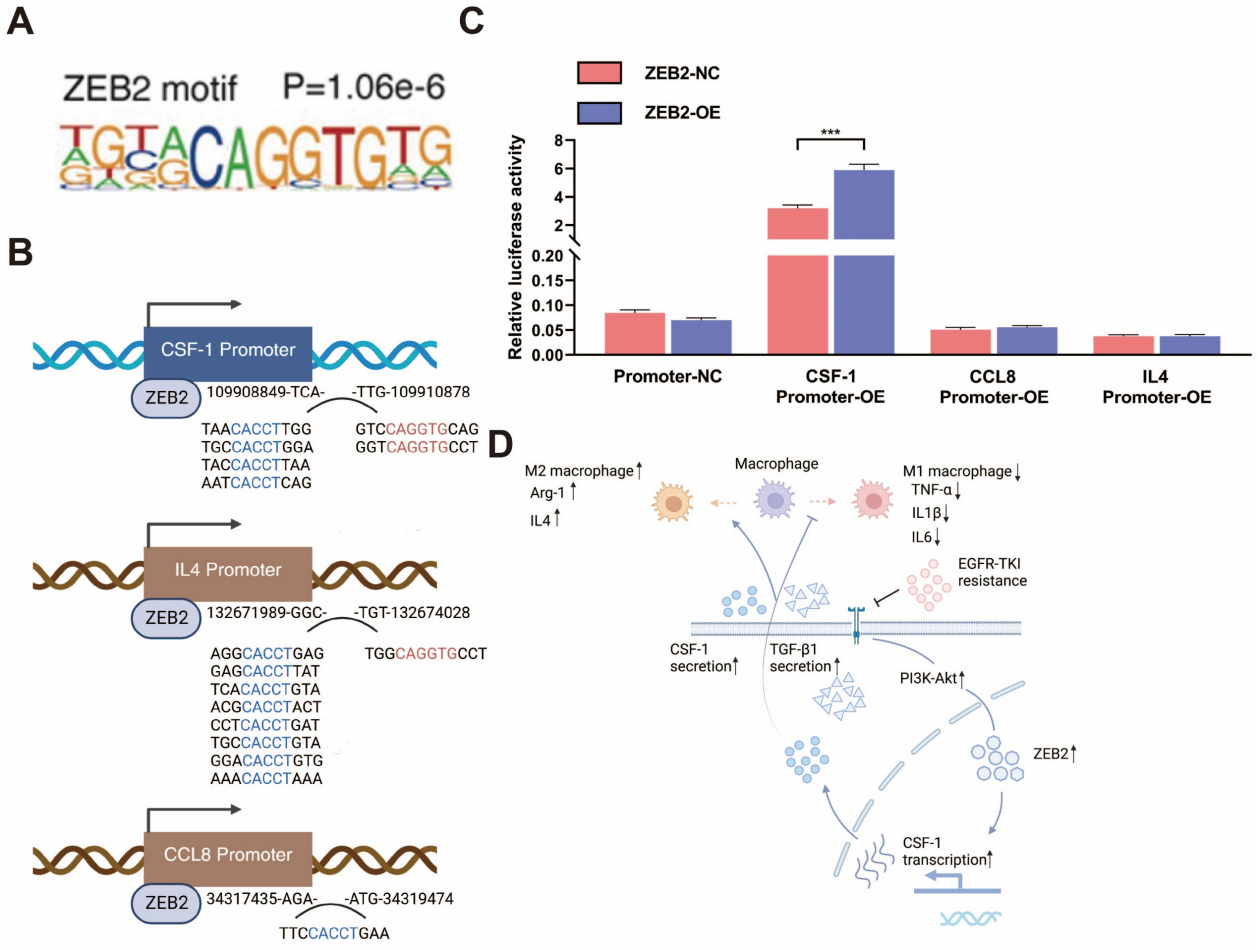
Mechanism underlying the modulating effect of ZEB2 on cytokine secretion. (A) The binding motif of ZEB2 quoted from previous research^[35]^; (B) Schematic illustration showing that the binding motif of ZEB2 and its palindromic sequence could match several regions of the potential promoter sequences of CSF-1, IL4, and CCL8; (C) Relative luciferase activity in 293T cells transfected with plasmids containing CSF-1 or IL4 or CCL8 promoter (GV534-Promoter-OE), a plasmid expressing ZEB2 (GV712-ZEB2-OE), and the corresponding empty vector; (D) Schematic illustration indicating that, in a PI3K-Akt-dependent manner, ZEB2 is upregulated following EGFR-TKI resistance. This upregulation promotes M2 polarizaiton and inhibits M1 polarization of TAMs by increasing the secretion of CSF-1 and TGF-β1 in NSCLC. ^***^P < 0.0005.

CSF-1 and IL4 are classic modulators of M2 polarization of TAMs, while TGF-β1 has been proved to be an anti-inflammatory biomarker. Moreover, it was verified in previous research that CCL8 could induce M2 polarization of macrophages^[48]^, while CXCL9 was associated with M1 polarization^[53]^. The upregulation of TGF-β1 by ZEB2 has been elucidated in hepatocellular carcinoma^[54]^. Furthermore, another research reported that ZEB2 participates in activating a positive feedback loop that enhances TGF-β1 production^[55]^. However, the underlying mechanism of ZEB2’s effect on CSF-1, IL4, and CCL8 had not been fully clarified. ZEB2, as a transcription factor, can directly bind the promotor regions of cytokines to regulate their expression; therefore, it is essential to assess this direct regulation of ZEB2 on these three cytokines. The binding motif of ZEB2 is presented in Figure 7A. The binding motif of ZEB2 and its palindromic sequence could match several regions of the potential promoter sequences of CSF-1, IL4, and CCL8 [Figure 7B]. We then investigated whether ZEB2 initiated the transcription of CSF-1, IL4, and CCL8 when bound to their promoter regions using dual luciferase reporter gene assay. The results of this experiment are presented in Figure 7C. When plasmid containing overexpressed ZEB2 was transfected, the transcription activity of CSF-1 promoter in 293T cells was markedly elevated, indicating that ZEB2 could bind to the promoter region of CSF-1 to initiate its transcription. However, the transcription activity of the IL4 promoter and CCL8 promoter showed no significant elevation after the transfection of the ZEB2-OE plasmid. Thus, these results verified that ZEB2 could induce CSF-1 secretion by directly binding to its promoter region. In conclusion, ZEB2 could promote M2 polarization and impede M1 polarization by elevating the secretion of CSF-1 and TGF-β1 [Figure 7D].

## DISCUSSION

The therapeutic strategy of NSCLC has been tremendously changed by ICI therapy^[56]^. However, multiple large-scale clinical trials pointed out that the benefit of ICI treatment in patients resistant to EGFR-TKI was controversial^[8,9]^. It was indicated in several studies that the immunosuppressive TME, characterized by the masking of tumor antigens and the proliferation of suppressive immune cells, significantly reduces the efficacy of ICI, thereby worsening the prognosis of patients dependent on these agents^[57-62]^. Hence, to maximize the clinical benefit of ICI in EGFR-driven NSCLC, especially NSCLC resistant to EGFR-TKIs, it is essential to investigate the mechanisms by which EGFR-TKI resistance contributes to the development of an immunosuppressive microenvironment. Previous work of our team elucidated the changes in the TME after the formation of osimertinib resistance^[63]^, and pointed out that during the usage of EGFR-TKIs, suppressive immune cells, such as MDSCs and M2 TAMs, gradually accumulated, which may lead to unsatisfactory responses to ICI therapy. Based on these studies, this research focused on the interaction between EGFR-TKI-resistant NSCLC tumor cells and TAMs, aiming to elucidate the inducement of M2 polarization and inhibition of M1 polarization of TAMs in TKI-resistant microenvironment. It was suggested in this research for the first time that EGFR-TKI resistance upregulated ZEB2 via PI3K-Akt signaling, whose overexpression induced the release of several cytokines, such as CSF-1 and TGF-β1, mediating M2 polarization and inhibiting M1 polarization of TAMs. Significantly, ZEB2 blockade could reverse the polarization of TAMs both in vitro and *in vivo*, which may contribute to rendering the immunosuppressive trend of the transformation of TME in EGFR-TKI-resistant NSCLC, serving as a potential target for therapeutic intervention synergistic to ICI therapy.

ZEB2 belongs to the zinc-finger E homeobox-binding family of transcription factors, interacting with activated SMADs^[64]^. It was elucidated in research more than 20 years ago that heterozygous mutation of ZEB2 was one of the etiologic causes of Mowat-Wilson syndrome (MWS)^[33,34,65]^, a neurocristopathy characterized by facial gestalt, intellectual disability, microcephaly, congenital heart defects, and Hirschsprung’s disease^[66,67]^. This correlation exhibits its crucial role in the embryonic development of neural and neural crest lineages. Furthermore, the critical role of ZEB2 in determining the fate of various immune cell types has recently garnered increasing attention from researchers, highlighting it as a topic worthy of further investigation. ZEB2 is essential for age-associated B cell accumulation and function by repressing MEF2B and Itgax, presenting as a powerful driver for B cell autoimmunity and a potential target for intervention in autoimmune disease^[35]^. ZEB2 can also mediate the presentation of both cytotoxic phenotype and B cell helper functions in a newly designated T cell subtype, “age-associated T helper (ThA) cells”, a distinct CXCR3^mid^CD4^+^ effector memory T cell population that expands in various autoimmune diseases^[36]^. Moreover, CD11c^+^ atypical B cells, indispensable in limiting recrudescent infection, depend on ZEB2 for recruitment and functioning^[37]^.

As a potent inducer of EMT, the role of ZEB2 has been elaborated in various cancers, such as breast carcinoma^[68]^, colorectal carcinoma^[69]^, gynecologic carcinoma^[70,71]^, and lymphoblastic/myeloblastic leukemia^[72,73]^. The relationship between ZEB2 and poor prognosis has been clarified in previous research. In colorectal carcinoma, a clinical study showed that ZEB2 upregulation is related to early recurrence and reduced survival^[38]^. Another research reported that ZEB2 induces peritoneal metastasis of high-grade serous ovarian carcinoma^[70]^. In a study on NSCLC, ZEB2 expression was upregulated in metastatic NSCLC tissues, while knockdown of this biomarker could hinder NSCLC cell migration and invasion^[74]^. Another aspect of research concerning ZEB2’s role in cancer was its function in resistance development. In colorectal carcinoma, ZEB2 was highly upregulated in a subgroup of quiescent/slow-cycling cells, inducing pCRAF/pASK1 elevation and then chemoresistance^[69]^. As to NSCLC, it was also elucidated in previous research that the PAX-ZEB2 axis could mediate metastasis and cisplatin resistance via the PI3K-Akt pathway, though the relationship between PI3K-Akt and ZEB2 was not fully clarified^[75]^. In our research, the expression of ZEB2 was increased in both EGFR-TKI-resistant NSCLC tumor cells and EGFR-TKI-resistant patients’ samples. These results demonstrated the close relationship between this EMT modulator and EGFR-TKI resistance formation in NSCLC.

The relationship between ZEB2 and multiple signaling pathways has already been discussed in previous studies. It was presented in a hematologic study that ZEB2 knockout in the bone marrow could lead to phenotypes resembling myeloproliferative disorders, which may be mediated by its downstream JAK-STAT and ERK pathways, according to KEGG analysis. This research confirmed ZEB2’s role in modulating cytokine signaling, while its regulatory effect on the PI3K-Akt pathway was not detected^[76]^. Based on the results of western blotting, qRT-PCR, and ELISA, ZEB2 was indeed correlated with cytokine secretion in our research, and the PI3K-Akt pathway was proved to be its upstream regulator, which may explain the observation in the previous hematologic research. In addition, it was clarified that epidermal growth factor (EGF) induced the upregulation of ZEB1 and ZEB2 via the Akt signaling pathway in gastric carcinoma^[77]^, which was consistent with the results of our research. Resistance to EGFR-targeted therapies could be categorized into primary or secondary resistance. Primary resistance, often caused by pre-existing mutations or alterations in key signaling pathways, limits the efficacy of EGFR-TKIs from the outset. In contrast, secondary resistance arises after an initial response to treatment, with tumors acquiring new mutations that enable them to bypass EGFR inhibition. At the molecular level, resistance to EGFR-targeted therapies can result from on-target mutations such as T790M and C797S, which impair drug binding, and off-target mechanisms, such as MET amplification and alterations in downstream signaling pathways, including PI3K-Akt and MAPK-ERK. These mutations activate compensatory signaling pathways that promote tumor survival and proliferation. In this background, our study focused on the PI3K-Akt pathway and its role in ZEB2 upregulation in EGFR-TKI-resistant NSCLC, contributing to the induction of M2 polarization in TAMs. This further exacerbated the suppressive TME, facilitating immune evasion and treatment resistance.

Several studies on lymphoblastic/myeloblastic leukemia have highlighted the role of ZEB2 in the proliferation and differentiation of immune cells from different perspectives. It has been suggested that miR-200c/141 overexpression, which is correlated with the exacerbation of T cell prolymphocytic leukemia, can downregulate ZEB2 and the TGF-β pathway, demonstrating a negative correlation between ZEB2 expression and T cell proliferation^[72]^. Though the relationship between ZEB2 and the TGF-β pathway was not clearly clarified in this study, another study on hepatocellular carcinoma complements this part. ZEB2 deubiquitination, which is facilitated by DGKG upregulation, increases TGF-β1 secretion, inducing regulatory T cell differentiation and immune evasion afterwards^[54]^. Consistent with these studies, ZEB2 upregulation is correlated with increasing TGF-β1 expression, which then induces M2 polarization and inhibits M1 polarization of TAMs as a canonical suppressive immune modulator in our research. In addition, CSF-1 and IL4, which are both classic M2 polarization inducers, are associated with ZEB2 upregulation for the first time in NSCLC in our research.

Transformation of the TME can be observed during the development of EGFR-TKI resistance. Although increasing CD8+ T cells were observed after EGFR-TKI treatment, this elevation was reversed after TKI resistance development in 21 cases of EGFR mutant NSCLC^[78]^. Additionally, the association between increasing CD8+ T cells and the efficacy of EGFR-TKI treatment was confirmed by single-cell RNA sequencing in another study^[31]^. As to CD4^+^ T cells, after the patient developed resistance to TKIs, such as gefitinib^[79]^ and ceritinib^[80]^, an increase in both the number and function of Tregs was observed. After gefitinib treatment, the cytotoxicity of NKs was enhanced via several mechanisms^[81,82]^. However, these changes were influenced by the efficacy of gefitinib^[28]^. According to previous work of our group, after osimertinib resistance developed, TAM infiltration increased in NSCLC, while M2 polarization was upregulated^[63]^. In current research, gefitinib-resistant NSCLC tumor cells could induce M2 polarization and inhibit M1 polarization via ZEB2 upregulation. In paired gefitinib-sensitive and -resistant patients’ samples, CD206 expression was increased after gefitinib resistance developed, while its expression was positively correlated with ZEB2 expression according to the results of IHC. Based on the CDX mouse model, ZEB2 knockout TKI-resistant NSCLC tumor cells could upregulate M1 polarization and impede M2 polarization compared to the control group. These findings from both in vitro and *in vivo* experiments not only confirm the conclusions of previous studies on EGFR-TKI resistance and M2 polarization of TAMs, but also elaborate on the role of ZEB2 in this process for the first time.

This study presents several limitations that warrant further investigation. First, although TAMs are the most abundant cell types in the TME and serve as key indicators of TME transformation, the role of other immune cell types, such as CD4^+^ T cells, CD8+ T cells, NK cells, and B cells, in the formation of a suppressive immune microenvironment were not comprehensively explored in the current study, and their interactions with ZEB2 require further examination to provide a more holistic understanding of the immune landscape in EGFR-TKI-resistant NSCLC. Second, the lack of a developed thymus leads to the absence of mature T cells in BALB/c nude mice. Though their innate immune system, including the monocyte-derived macrophages, remains intact, it may still be affected by the overall immune deficiency. Hence, the impact of ZEB2 on the whole landscape of TME should be verified in future studies using humanized or immunocompetent mouse models. Our team is currently utilizing the Cre-LoxP system to generate a transgenic EGFR-mutant NSCLC mouse model, which will be used to assess ZEB2 inhibition in the immunocompetent mouse model and to explore its potential synergistic effect with immunotherapy. These efforts are ongoing and will be addressed in future work. Furthermore, the current study focused exclusively on first-generation EGFR-TKIs, such as gefitinib and erlotinib, and did not investigate third-generation EGFR-TKI resistance, such as osimertinib. Lastly, the subcellular localization of ZEB2 and CD206 could be further explored with in situ multiplex immunofluorescence; however, IHC staining was employed instead in the study. We acknowledge these limitations and are committed to addressing them in future studies to deepen our understanding of the role of ZEB2 in the immune microenvironment of EGFR-TKI-resistant NSCLC.

In conclusion, in EGFR-TKI-resistant NSCLC, the activation of the PI3K-Akt cascade drove a marked rise in ZEB2 expression. The elevated ZEB2 increased CSF-1 and TGF-β1 release, steering macrophages toward an M2 phenotype while impeding M1 polarization. Accordingly, suppressing ZEB2 had the potential to reshape the TME and enhance the effectiveness of ICIs once EGFR-TKI resistance had emerged.

## DECLARATIONS

### Availability of data and materials

The data supporting the findings of this study are available within this Article and its Supplementary Information. Further data are available from the corresponding authors upon reasonable request.

### Copyright

© The Author(s) 2025.
