# Supplementary material for: ZEB2 upregulation modulates the polarization of TAMs toward the immunosuppressive state in EGFR-TKI-resistant NSCLC
Source: Cancer Drug Resist. 2025 May 29;8:25. doi: 10.20517/cdr.2024.206 (PMC12159605; doi:10.20517/cdr.2024.206)
Supplement: Supplementary file 1 [file cdr-8-25-SupplementaryMaterials.pdf]

Supplementary Materials

ZEB2 upregulation modulates the polarization of TAMs toward the immunosuppressive state in EGFR-TKI-resistant NSCLC

Yunhuan Liu[1,#], Yong Yu[3,#], Congli Hu[1,#], Minlin Jiang[1,#], Chao Zhao[1], Xuefei Li[1], Lei Cheng[1], Caicun Zhou[2]

[1]Department of oncology, Shanghai Pulmonary Hospital Affiliated to Tongji University, Shanghai 200433, China.

[2]Department of oncology, Shanghai East Hospital Affiliated to Tongji University, No.1800 Yuntai Road, Pudong New District, Shanghai 200120, China.

[3]Department of Radiology, Yancheng TCM Hospital Affiliated to Nanjing University of Chinese Medicine, Yancheng 224002, Jiangsu, China.

[#]Authors contributed equally.

Correspondence to: Dr. Zhou Caicun, Department of oncology, Shanghai East Hospital Affiliated to Tongji University, No.1800 Yuntai Road, Pudong New District, Shanghai 200120, China. E-mail: caicunzhoudr@163.com; Dr. Cheng Lei, Department of oncology, Shanghai Pulmonary Hospital Affiliated to Tongji University, No.507 Zhengmin Road, Yangpu District, Shanghai 200433, China. E-mail: chenglei_2008@126.com

Supplementary Table 1. Basic characteristic information of patients recruited in this research

Patient	Gender	Age	Histology	Primary tumor size (mm)	Metastasis	TMN
1	M	50	Adenocarcinoma	34.1	Brain	T3N0M1b
2	M	73	Adenocarcinoma	47.5<br>23.3	Contralateral lung	T2bN3M1a
3	F	58	Adenocarcinoma	76		T4N2Mx
4	F	54	Adenocarcinoma		Contralateral lung	T4N0M1a
5	F	77	Adenocarcinoma	60.5	Bone<br>Pleura	T4N2M1c
6	F	69	Adenocarcinoma	46		T2N2M0
7	F	68	Adenocarcinoma	61.8<br>17.6	Contralateral lung<br>Bone	T3N2M1c
8	M	71	Adenocarcinoma		Contralateral lung	T4N2M1a
9	M	41	Adenocarcinoma	42.5	Pleura	T4N4M1a
10	F	49	Adenocarcinoma	73	Rib	T4N3M1c

Supplementary Table 2. Specific primers used in this research

Target genes	Forward	Reverse
IL12	AGGGCCGTCAGCAACATG	TCTTCAGAAGTGCAAGGGTAAAATTC
IL6	AAGCCAGAGCTGTGCAGATGAGTA	TGTCCTGCAGCCACTGGTTC
IL1β	AGCTACGAATCTCCGACCAC	CGTTATCCCATGTGTCGAAGAA
TNF-α	CAGAGGGAAGAGTTCCCCAG	CCTGGTCTGGTAGGAGACG
IL4	ATGGGTCTCACCTCCCAACT	GATGTCTGTTACGGTCAACTCG
Arg-1	CTGGCAAGGTGGCAGAAGTC	ATGGCCAGAGATGCTTCCAA
ZEB2	GCGATGGTCATGCAGTCAG	CAGGTGGCAGGTCATTTTCTT
IL-10	GCTGTCATCGATTTCTTCCC	CTCATGGCTTTGTAGATGCCT
CCL2	CTTCTGTGCCTGCTGCTCATA	CTTTGGGACACTTGCTGCTG
CCL5	ACCAGTGGCAAGTGCTCCAAC	CAGCCGGGAGTCATACAGGA
CCL8	TGGAGAGCTACACAAGAATCACC	TGGTCC AGATGCTTCATGGAA
CXCL1	CAAACCGAAGTCATAGCCACAC	GGATTTGTCACTGTTCAGCATCTT
CXCL9	TCTTGCTGGTTCTGATTGGAGTG	TAGTCCCTTGGTTGGTGCTGAT
CXCL10	GGCCATCAAGAATTTACTGAAAGCA	TCTGTGTGGTCCATCCTTGGAA
CXCL11	CCTTGGCTGTGATATTGTGTGCTA	CCTATGCAAAGACAGCGTCCTC
CSF-1	GATGGAGACCTCGTGCCAAATTA	TGTTATCTCTGAAGCGCATGGTG
CCL20	TGCTGTACCAAGAGTTTGCTC	CGCACACAGACAACTTTTTCTTT
CXCL8	TCTGCTAGCCAGGATCCACA	TGCTTCCACATGTCCTCACA
GAPDH	GCACCGTCAAGGCTGAGAAC	TGGTGAAGACGCCAGTGGA

Supplementary Table 3. LogFC and adjusted P value of 17 common most highly upregulating DEGs in HCC827 before and after erlotinib resistance

Gene Name	Log FC	Adjusted P value
CSDC2	10.74173	9.39E-14
ZEB2	9.0393	4.21E-18
SERPINE1	8.979462	6.72E-10
AK5	8.799701	6.28E-18
TENM2	8.667625	0.000223
ANO2	8.437445	2.61E-05
APCDD1L	8.279027	0.000576
RNF182	8.251074	2.29E-10
TWIST1	8.047293	0.002829
CHST9	7.926356	5.80E-17
RGS4	7.884844	3.46E-08
ACSM5	7.797203	1.53E-09
CDK15	7.72232	0.001702
FAM43B	7.542032	2.15E-07
MMP16	7.259201	3.84E-06
CNN1	7.150792	3.60E-05
ALPK2	7.145911	6.44E-13

Supplementary Table 4. LogFC and adjusted P value of 17 common most highly upregulating DEGs in HCC4006 before and after erlotinib resistance

Gene Name	Log FC	Adjusted P value
TENM2	14.63483	8.34E-18
CNN1	14.28409	1.59E-28
APCDD1L	10.50306	8.71E-13
CHST9	10.47656	1.31E-13
RGS4	10.31609	8.82E-14
ALPK2	10.05742	2.06E-12
ACSM5	9.868678	7.87E-13
CDK15	9.746589	1.18E-11
RNF182	9.647935	2.05E-12
FAM43B	8.814982	2.45E-10
ZEB2	8.454783	8.60E-48
CSDC2	8.279258	8.39E-17
ANO2	8.193553	4.69E-09
AK5	8.191282	4.93E-18
MMP16	8.019539	2.67E-14
SERPINE1	7.665034	5.52E-24
TWIST1	7.464798	2.99E-58

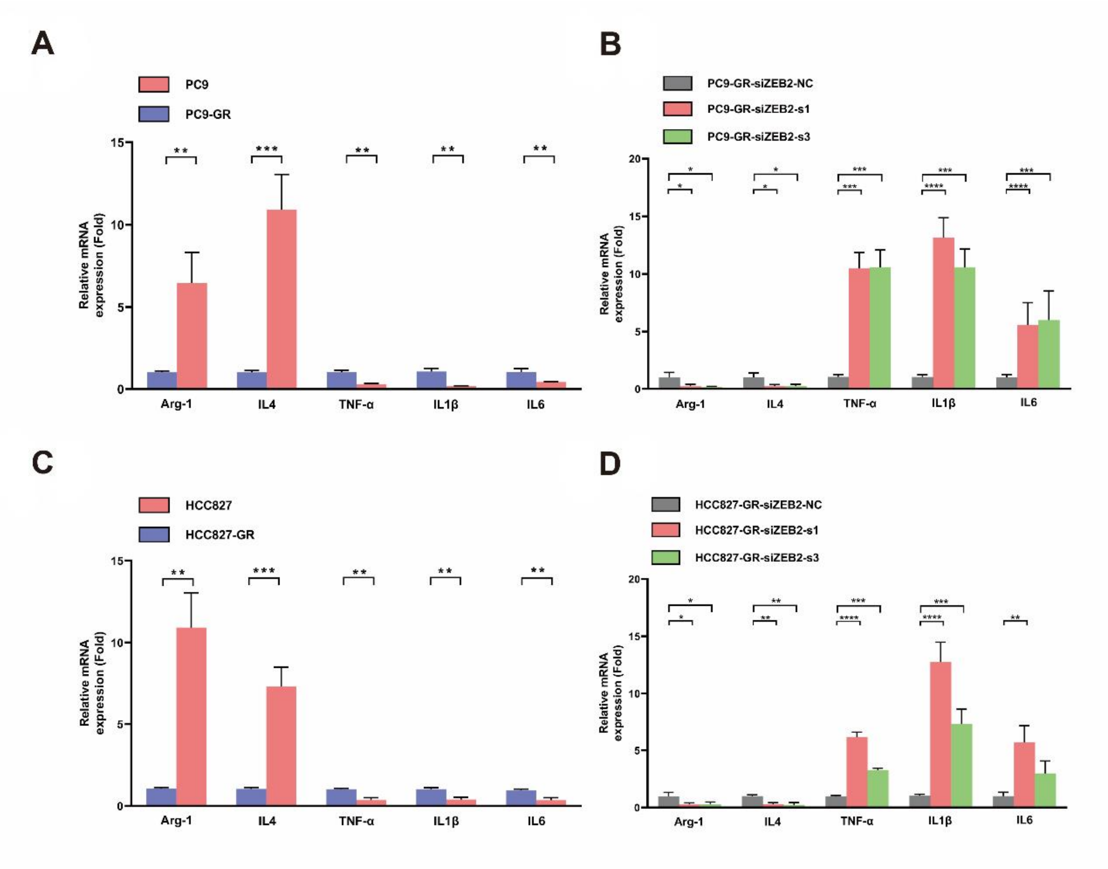

Supplementary Figure 1. Effect of ZEB2 in EGFR-TKI resistance on M1/M2 polarization of primary human TAMs in NSCLC. (A) qRT-PCR analysis showing the induction of M1/M2 polarization in human TAMs by conditional media (CM) from EGFR-TKI sensitive and resistant PC9 cells; (B) Effect of ZEB2 knockdown on M1/M2 polarization of human TAMs in PC9-GR; (C) qRT-PCR analysis showing the induction of M1/M2 polarization in human TAMs by CM from EGFR-TKI sensitive and resistant HCC827 cells; (D) Effect of ZEB2 knockdown on M1/M2 polarization of human TAMs in HCC827-GR.

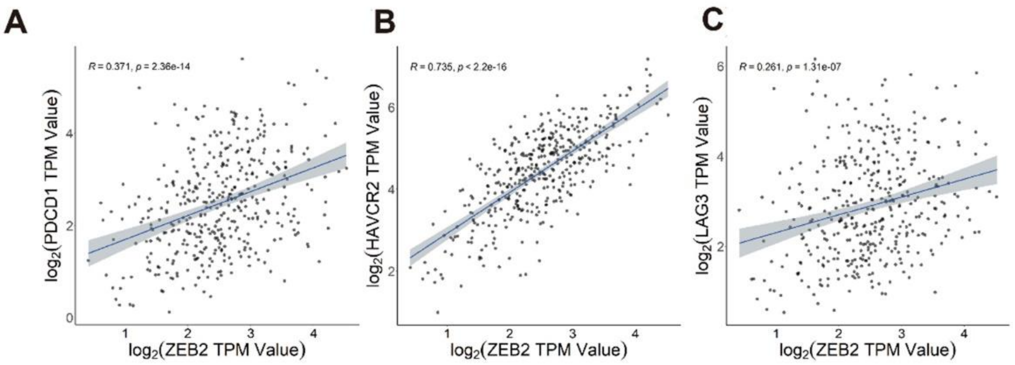

Supplementary Figure 2. The correlations between ZEB2 expression and biomarkers of T cell dysfunction. (A-C) Pearson correlations between the expression of ZEB2 and the expression of PDCD1 (R = 0.371), HAVCR2(TIM-3) (R = 0.735) and LAG3 (R = 0.261) in LUAD cohort from TCGA database. With correlation coefficient > 0.6, ZEB2 was strongly correlated with HAVCR2(TIM-3), which was an immune checkpoint receptor that played a crucial role in regulating immune responses by promoting T cell exhaustion and limiting immune activation in NSCLC.

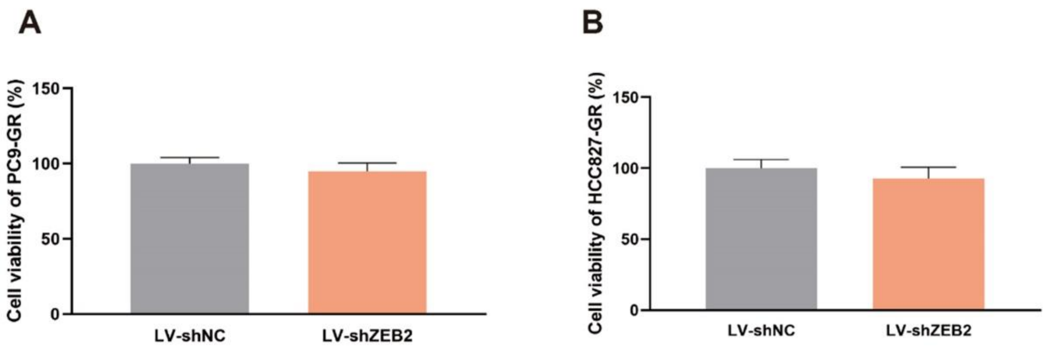

Supplementary Figure 3. Effect of ZEB2 knockdown on the viability of PC9-GR and HCC827-GR cells assessed by CCK-8 assay. (A) The effect of ZEB2 knockdown on the cell viability of PC9-GR cells; (B) The effect of ZEB2 knockdown on the cell viability of HCC827-GR cells. The data represented the average results from three independent experiments. The statistical significance was determined by comparing the LV-shZEB2 groups with the LV-shNC groups.
